# The Strengthening and Toughening of Biodegradable Poly (Lactic Acid) Using the SiO_2_-PBA Core–Shell Nanoparticle

**DOI:** 10.3390/ma12162510

**Published:** 2019-08-07

**Authors:** Hailing He, Yuezhao Pang, Zhiwei Duan, Na Luo, Zhenqing Wang

**Affiliations:** 1 College of Aerospace and Civil Engineering, Harbin Engineering University, Harbin 150001, China; 2 Institute of Fluid Physics, China Academy of Engineering Physics, Mianyang 621900, Sichuan, China

**Keywords:** core–shell nanoparticle, poly (lactic acid), mechanical properties, finite element model

## Abstract

The balance of strengthening and toughening of poly (lactic acid) (PLA) has been an intractable challenge of PLA nanocomposite development for many years. In this paper, core–shell nanoparticles consisting of a silica rigid core and poly (butyl acrylate) (PBA) flexible shell were incorporated to achieve the simultaneous enhancement of the strength and toughness of PLA. The effect of core–shell nanoparticles on the tensile, flexural and Charpy impact properties of PLA nanocomposite were experimentally investigated. Scanning electron microscopy (SEM) and small-angle X-ray scattering (SAXS) measurements were performed to investigate the toughening mechanisms of nanocomposites. The experimental results showed that the addition of core–shell nanoparticles can improve the stiffness and strength of PLA. Meanwhile, its elongation at break, tensile toughness and impact resistance were enhanced simultaneously. These observations can be attributed to the cavitation of the flexible shell in core–shell nanoparticles and the resultant shear yielding of the matrix. In addition, a three-dimensional finite element model was also proposed to illustrate the damage processes of core–shell nanoparticle-reinforced polymer composites. It was found that, compared with the experimental performance, the proposed micromechanical model is favorable to illustrate the mechanical behavior of nanocomposites.

## 1. Introduction

The environmental and resource problems induced by the excessive use of petroleum-based plastics have generated immense interest in the development of biodegradable polymers [1]. Poly (lactic acid) (PLA), produced from natural resources such as corn starch and sugar cane, is the most promising biopolymer, profiting from its high strength, stiffness, transparency and thermal plasticity [2,3]. Recently, PLA has been used in many fields, such as biomedical devices, vehicle interiors, and food/beverage packages [4]. However, the inherent brittleness of PLA impedes its larger-scale application [5]. Introducing rubber or rigid filler is a frequently-used strategy to overcome the inherent brittleness of PLA. For example, the incorporation of natural rubber can significantly improve the toughness of PLA, but this results in the dramatic decrease of strength and modulus [6,7]. On the contrary, rigid fillers such as nanometer calcium carbonate, clay or talc nanotubes can enhance the strength and modulus of PLA while its toughness increases slightly, even under the situation of elongation at break declining [8,9,10]. Consequently, Thitsartarn et al. [11] proposed a core–shell nanoparticle consisting of rigid and rubbery segments to simultaneously enhance the strength and toughness of epoxy. Subsequently, Liu et al. [12] synthetized a nanosilica-rubber core–shell particle via simple ring-opening polymerization. The copolymer of caprolactone and meso-lactide was grafted on a rigid silica inner core to improve the facture toughness of epoxy. The strength and elongation at break of the epoxy composite were enhanced as well. Thus, it can be seen that this newly-developed core–shell nanofiller can be used for the strengthening and toughening of epoxy nanocomposites. Despite these developments, there have been no efforts to simultaneously increase the toughness and strength of PLA using this rigid core–soft-shell nanoparticle. Therefore, in this study, a core–shell nanofiller consisting of a rigid silica core and flexible rubber shell was applied to improve the mechanical properties of PLA biopolymer.

Experimental investigations of core–shell nanoparticle-reinforced composites have been performed, but their failure process and mechanism could not be completely revealed in the experimental process. Moreover, full optimization experiments into the effect of complex core–shell filler on the mechanical behavior of nanocomposites are unfeasible and expensive. Therefore, a numerical modeling based on the microstructure of core–shell nanocomposites is required to predict the mechanical behavior of composites. Recently, a few numerical investigations of particle-reinforced composites have been performed. For example, Williams et al. [13] compared the different mechanical behaviors of particle-reinforced metal matrix composites with a perfect interface and debonding interface based on the cohesive zone model (CZM). Meng and Wang [14] investigated the debonding failure process of SiC particle-reinforced Al matrix composites using a three-dimensional micromechanical model. However, the microstructural model of core–shell particle-reinforced composites has been not presented in the published references. Besides this, pervious micromechanical models only focused on the debonding damage of the interface between particle and matrix and did not account for the matrix cracking damage. As Cho et al. [15] reported, both matrix cracking and particle/matrix debonding led to the nonlinear behavior of the stress–strain curves. Tsui et al. [16] also found that microcracks of the matrix could be initiated when the propagation rate of interface debonding declined at a specific strain via in situ SEM experiment. The matrix cracking has been verified experimentally: however, its detailed failure mechanism based on the numerical simulation has barely been reported. Therefore, the damage processes of both interfacial debonding and matrix cracking are considered in our micromechanical model.

In this paper, the core–shell nanofiller was applied to improve the strength and toughness of PLA nanocomposite, in which nano-silica and Poly (butyl acrylate) (PBA) were designed as the rigid inner core and flexible outer shell of the core–shell filler, respectively. The effect of core–shell nanoparticles on the tensile, flexural and Charpy impact properties of PLA nanocomposite were experimentally investigated. For comparison, the properties of composites incorporated by raw and silanized silica were studied as well. Further, to understand the effect of core–shell nanoparticles on the mechanical behavior and failure mechanism of PLA-based composites, the experimental results were analyzed numerically using the finite element method (FEM). Both the interfacial debonding and matrix damage were considered in the three-dimensional micromechanical model based on the cohesive zone model and ductile damage model, respectively.

## 2. Materials and Methods

### 2.1. Materials

Poly (lactic acid) (3051D, 96.5% of l-lactide, *M*_w_:160 kDa, Polydispersity:1.7) was purchased from Natureworks (Blair, NE, USA). Nanosilica (Aerosil 380) white powder with a specific surface area of 380 ± 30 m^2^/g was obtained from Evonik Industries AG. (Essen, Germany). Butyl acrylate (n-BA, >99%), 2-bromoisobutyryl bromide (BiB, 97%), ethyl-2-bromoisobutyrate (EBiB, 98%), cysteamine (>95%), and triphenylphosphine (PPh_3_ >99%) were purchased from Aldrich Chemical Reagents Co. (Shanghai, China) and used as received. Three-Aminopropyltriethoxysilane (APTES, >99%), Iron (III) chloride hexahydrate (FeCl_3_·6H_2_O, >99%), triethylamine (TEA, >99.0%), ascorbic acid (VC, >99.7%), N,N-dimethylformamide (DMF; analytical reagent), toluene (analytical reagent), tetrahydrofuran (THF; analytical reagent) and other chemicals were obtained from Tianjin Chemical Agent Company (Tianjin, China) and used as received unless otherwise mentioned.

### 2.2. Synthesis of Core–Shell Nanoparticles

1. Synthesis of amino-functionalized silica nanoparticles (SiO_2_-NH_2_)

Silica nanoparticles (1.0 g) and dry toluene (30.0 mL) were added to a 100 mL flask to form a homogeneous solution. After evacuating air with N_2_ for 30 min, APTES (2 mL) was added dropwise to the above mixture. Then, the reaction mixture was stirred for 16 h at 95 °C in an oil bath. After cooling down to room temperature, the functionalized silica nanoparticles were washed with toluene three times, collected by centrifugation, and then dried under vacuum at 60 °C for 24 h.

2. Synthesis of initiator-immobilized silica nanoparticles (SiO_2_-Br)

SiO_2_-NH_2_ (1.0 g), trimethylamine (2 mL) and toluene (15 mL) were placed in a flask under magnetic stirring and a nitrogen atmosphere. After the solution was cooled to 0 °C in an ice water bath, the mixture of 2-bromoisobutyryl bromide (2 mL) and toluene (5 mL) was added dropwise to the above solution. The reaction mixture was stirred for 3 h at 0 °C and 12 h at room temperature. The obtained mixture was washed three times with toluene, precipitated by centrifugation and then dried under vacuum at 60 °C for 24 h.

3. Synthesis of core–shell nanoparticles (SiO_2_-PBA-Br)

FeCl_3_·6H_2_O (58 mg), PPh_3_ (424 mg), SiO_2_-Br (0.5 g), DMF (20 mL), n-BA (20 mL), and EBiB initiator (90 µL) were added to a glass vial and sonicated for 10 min to form the homogeneous mixture. After the vial was sealed with a rubber stopper, the solution of VC (1.056 g) dissolved in the DMF (5 mL) was injected into the vial. Then, the sealed glass vial was placed in an oil bath at 90 °C to polymerize for 8 h. The synthesized product was washed five times with THF and dried under vacuum to a constant weight.

4. Amination of terminal group of core–shell nanoparticles (SiO_2_-PBA-NH_2_)

SiO_2_-PBA-Br (1.0 g), trimethylamine (0.02 mL) and cysteamine (12 mg) were dissolved in THF (20 mL) and stirred overnight at room temperature. The product was washed and precipitated in an excess mixture of methanol/water (*v*:*v* = 1:1).

### 2.3. Preparation of PLA-Based Nanocomposite

The neat PLA was dried in a vacuum oven for 8 h at 60 °C before use. The dried PLA pellets were grinded using a small crusher (27000 r/min, Yongkang, China) for 10 s and then sieved using a 50-mesh screen to obtain the PLA powder. Subsequently, the PLA powder and nanoparticles were added to the planetary ball mill (QM-3SP2, Nanjing, China) to disperse the nanoparticles uniformly at 600 r/min for 120 min. The obtained mixture were filled in the steel mould and then placed on the hot press for 5 min at 170 °C to melt the PLA resin. Afterwards, mold compression was conducted for 3 min under a pressure of 10 MPa to form the specimens. Control samples (denoted as Neat PLA) were also likewise prepared. The nanocomposite modified by raw silica (SiO_2_), silanized silica (SiO_2_-NH_2_) and core–shell nanoparticle (SiO_2_-PBA-NH_2_) were denoted as PLA/SiO_2_, PLA/SiO_2_-NH_2_ and PLA/SiO_2_-PBA-NH_2_, respectively. The content of various fillers is 1 wt.% of the total composite composition.

### 2.4. Characterization of Nanoparticle and Composites

The differential scanning calorimetry (DSC) measurement was conducted under N_2_ using a NETZSCH DSC 200F3 (NETZSCH-Gerätebau GmbH, Selb, Germany). The samples were first heated from 25 °C to 200 °C at a heating rate of 20 °C/min and held at 200 °C for 5 min to erase the thermal history. Subsequently, the temperature was cooled down to 0 °C at a rate of 20 °C/min and maintained for 2 min. Then, it was heated to 200 °C again at the rate of 10 °C/min. From the DSC thermograms, the glass transition temperature ($T_{g}$), melting temperature ($T_{m}$), and experimental melting enthalpy ($\Delta H_{m}$) were determined. The degree of crystallinity ($x_{c}$) can be calculated from the melting enthalpy by the equation$x_{c}=\left(\Delta H_{m}/\Delta H_{m}^{0}\right)÷w\times 100$, where the $\Delta H_{m}^{0}$ is the melting enthalpy of 100% crystalline PLA taken as 93 J/g [17] and w is the weight fraction of PLA. The tensile tests were performed using an Instron 5500R testing machine (Instron®, Norwood, MA, USA) at a crosshead speed of 2 mm/min according to the ASTM Standard D638. The dimensions of the dog-bone-shaped specimen are 150 × 10 × 4 mm^3^. The flexural properties were measured according to ASTM D790 with specimen dimensions of 80 × 15 × 6 mm^3^ using a Zwick/Roell Z010 machine (Zwick/Roell, Ulm, Germany). The Charpy impact strength of the notched PLA nanocomposite with dimensions of 50 × 10 × 5 mm^3^ was determined according to the ASTM D6110. The fracture surface of the tensile sample was observed using scanning electron microscopy (SEM) (JEOL JSM 6390) (JEOL Ltd. Tokyo, Japan). Small-angle X-ray scattering (SAXS) measurements were performed on an Anton Paar SAXSpoint 2.0 (Anton Paar GmbH, Graz, Austria) at 50 kV with a Cu Kα radiation of 0.15418 nm. The two-dimensional scattering patterns of SAXS were collected and the background scattering was subtracted.

**Figure 1:**
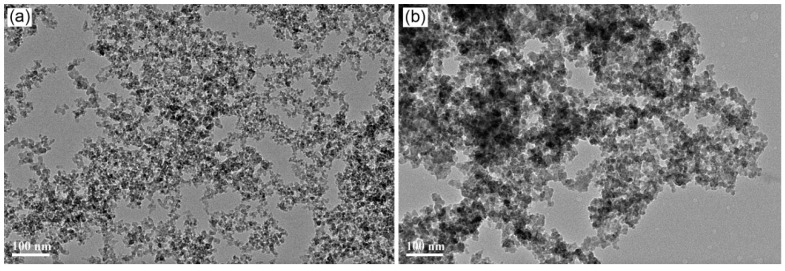
Transmission electron microscopy (TEM) images of (**a**) the raw SiO2 nanoparticle, (**b**) the SiO2-PBA-NH_2_ core–shell nanoparticle.

**Figure 2:**
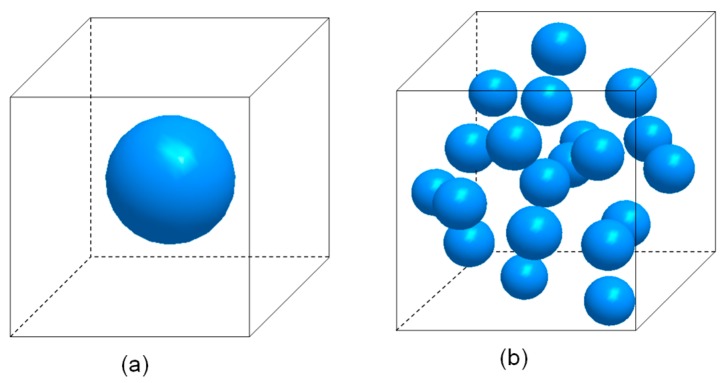
Representative microstructure of raw silica/poly (lactic acid) (PLA): (**a**) single particle, (**b**) multi-particle.

The characterization of the flexible polymer shell being successfully grafted on the silica surface is presented in the Supplementary Materials. The morphology and size of the raw silica and core–shell nanoparticles were investigated using a transmission electron microscope (TEM) (JEOL2100F) (JEOL Ltd. Tokyo, Japan) operated at 200 kV. The dilute solutions of the dispersed nanoparticles were sonicated for 10 min and then dropped onto a copper grid. Figure 1 shows the morphologies of the raw nano-silica and SiO_2_-PBA-NH_2_ core–shell nanoparticle. As presented in Figure 1, the size of the nanoparticle becomes bigger after surface copolymerization grafting. The average particle size of the raw nano-silica is about 11.3 nm. After grafting with butyl acrylate copolymer, the average particle size of the core–shell is about 19.3 nm. Moreover, from Figure 2, the border between the core–shell particles became more blurred than that of raw silica, which has a light polymer shell.

## 3. Finite Element Modelling

### 3.1. Representive Volume Element (RVE)

The three-dimensional microstructural models of both single-particle and multi-particle constellations are constructed to capture the mechanical behavior and failure process of the nanoparticle-reinforced PLA composites. The multi-particle model, of which the particles were distributed randomly. is more representative of a real material. The simplified single-particle model is presented to distinctly illustrate the local behavior of composites during progressive damage. In these models, both interfacial debonding and matrix damage were considered. 

The representative volume element (RVE) of the raw silica-reinforced nanocomposites are shown in Figure 2. One spherical rigid particle is placed at the centroid of a cube in the single-particle model (Figure 2a). The multi-particle model, containing a random dispersion of 20 non-overlapping identical spheres, is shown in Figure 2b. The random particles were generated using a constrained adsorption algorithm [18]. Subsequently, the matrix and particles are meshed with the tetrahedral element, and the zero-thickness 6-node three-dimensional cohesive element (COH3D6) is inserted between the particles and matrix to simulate the interfacial debonding. The numbers of elements of the single-particle and multi-particle model are approximately 197,400 and 371,300, respectively.

**Figure 3:**
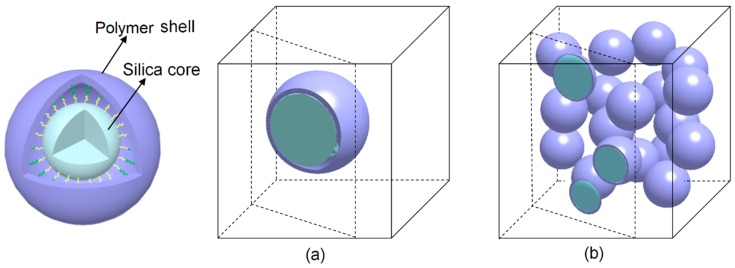
Representative microstructure of core–shell nanofiller-reinforced composite: (**a**) single particle, (**b**) multi-particle.

The single-particle and multi-particle models of the core–shell nanoparticle-reinforced PLA composite are presented in Figure 3a,b, respectively. The core–shell nanoparticle consists of a silica core and polymer shell. The covalent bonds between the rigid core and flexible shell are substituted by their intact bonding in the RVE models. In addition, the interface between the polymer shell and PLA matrix is introduced to capture the cavitation occurring within the soft polymer shell at the poles of the particle [19]. The numbers of elements of the single-particle and multi-particle model are approximately 128,700 and 311,900 tetrahedral elements, respectively. Moreover, the zero-thickness 6-node three-dimensional cohesive element (COH3D6) is inserted between the polymer shell and matrix to simulate the cavitation of the polymer shell.

### 3.2. Periodic Boundary Conditions

To capture the behavior of a truly periodic microstructure, periodic boundary conditions are applied to the models. When the axes of coordinates $x_{1},\;x_{2},\;x_{3}$ are aligned with the edges of the cell and the origin is placed at a corner of the cell, the periodic boundary condition can be expressed as a function of the displacement vector ***u*** as in [20]:

(1) $$\begin{array}{l} u(x_{1},x_{2},0)-u_{3}=u(x_{1},x_{2},L)\\ u(x_{1},0,x_{3})-u_{2}=u(x_{1},L,x_{3})\\ u(0,x_{2},x_{3})-u_{1}=u(L,x_{2},x_{3}) \end{array}$$

where $u_{1},u_{2},u_{3}$ depend on the particular loading applied on the unit cell. In this study, we mainly investigate the uniaxial tensile behavior of particle reinforce composite. The tensile load of the $x_{2}$ direction is obtained with $u_{3}=(0,0,u_{3})$, $u_{1}=(u_{1},0,0)$ and $u_{2}=(0,u,0)$, and $u_{1},u_{3}$ can be calculated from the conditions:

(2) $$\left\{ \begin{array}{l} \int \sigma_{1}d\Omega =0\;\mathrm{on}\;x_{1}=0\\ \int \sigma_{3}d\Omega =0\;\mathrm{on}\;x_{3}=0 \end{array}\right.$$

where $\sigma_{1},\;\sigma_{3}$ are the normal tractions in the planes of $x_{1}=0,\;x_{3}=0$, respectively, and Ω is the cross-section of each face in RVE.

The material models and parameters of the inclusion, matrix and interface are shown in the Supplementary Materials.

## 4. Experimental Results and Discussion

### 4.1. Thermal Properties

**Figure 4:**
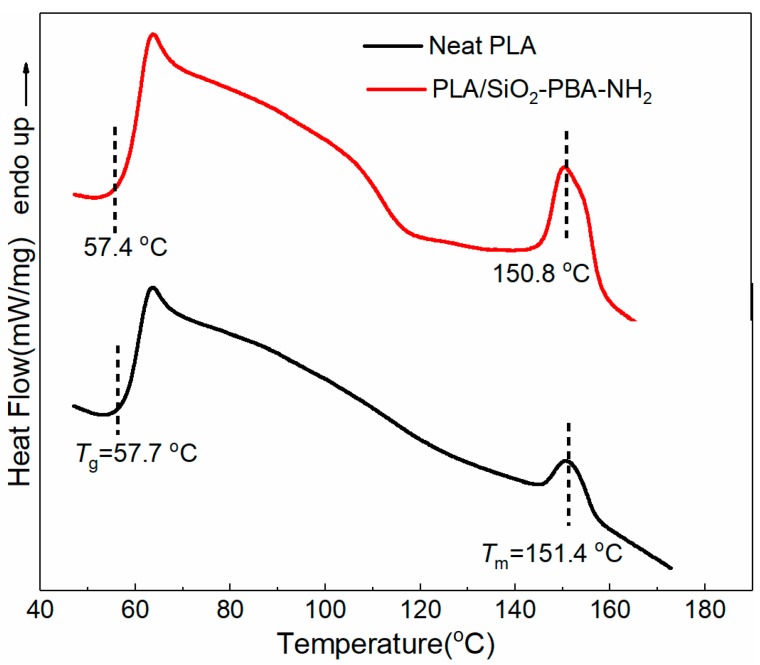
Differential scanning colorimetry (DSC) thermograms of neat PLA and PLA/SiO2-PBA-NH2 nanocomposite.

The effect of core–shell nanoparticles on the thermal properties and crystallization behaviors of neat PLA and PLA/core–shell nanocomposite are shown in Figure 4. The DSC thermogram shows that the glass transition and melting temperature of neat PLA are 57.7 °C and 151.4 °C, respectively. The addition of SiO_2_-PBA-NH_2_ core–shell nanoparticles exhibited little change, at 57.4 °C and 150.8 °C, respectively. Besides this, the crystallinity of neat PLA was derived from the DSC graphs of just 4.2%, which may be ascribed to the fact that poly (L-lactide) (PLLA) is semi-crystalline polymer. Its crystallization is particularly difficult unless it is induced by stretching in the fabrication process [10]. Notably, with the incorporation of a1 wt.% core–shell nanoparticles to PLA, the crystallinity of the PLA composite significantly increased to 8.6%, which can be ascribed to the fact that the core–shell nanoparticle can be used as a nucleation agent and thus induce the heterogenous crystallization of PLA more effectively. Similar results were also reported by Nofar et al. [21].

### 4.2. Mechanical Properties of Nanocomposites

**Figure 5:**
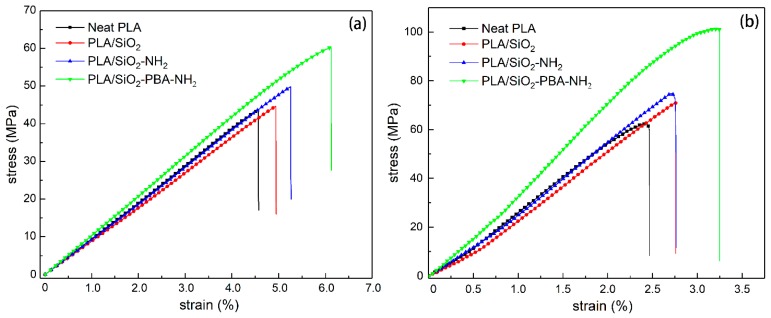
(**a**) Tensile and (**b**) flexural stress–strain curves of neat PLA and PLA nanocomposites.

The tensile and flexural stress–strain curves of neat PLA and nanoparticle-reinforced PLA composites are shown in Figure 5, and associated properties are tabulated in Table 1. The toughness was calculated by integrating the tensile stress–strain curves. It can be seen that the neat PLA and nanocomposites exhibit brittle fracture behaviors. Notably, as can be seen in Figure 5a,b, both the strength and failure strain of PLA significantly improve as the core–shell nanoparticles are introcuced. As can be seen in Table 1, in comparison with neat PLA, the tensile strength of PLA/SiO_2_-NH_2_ and PLA/SiO_2_-PBA-NH_2_ are enhanced by 17.6% and 36.9%, respectively, which are much higher than that of PLA/SiO_2_ of 4.2%. This could be attributed to the perfect interfacial interactions between the nanofillers and matrix to promote effective stress transfer. Moreover, the core–shell nanoparticle also has a significant effect on improving the ductility of PLA, increasing its elongation at break by 25.9%.

As can be seen in Figure 5b and Table 1, the neat PLA and PLA-based nanocomposites have superior flexural properties; the flexural strength and modulus of neat PLA are 63.3 MPa and 2.42 GPa, respectively. The addition of nano-SiO_2_ slightly increased the flexural strength to 71.2 MPa and reduced the flexural modulus to 2.28 GPa. The low loading of pristine SiO_2_ (1 wt. %) results in less enhancement of the mechanical properties of the polymer matrix, which can be concluded from many other works [22]. Furthermore, the SiO_2_-NH_2_ nanoparticle modified by the APTES coupling agent can moderately improve the flexural strength—by 18.5%—and the modulus was only marginally reduced. Notably, the incorporation of SiO_2_-PBA-NH_2_ core–shell nanoparticle significantly increased the flexural strength and modulus by 61.8% and 33.9% compared to that of neat PLA. Accordingly, the chemical bonding between the particle and matrix via the amidation reaction of the amino group on the core–shell surface and the carboxyl functionality of PLA can decrease the debonding of particles and promote the load transfer [23,24].

**Figure 6:**
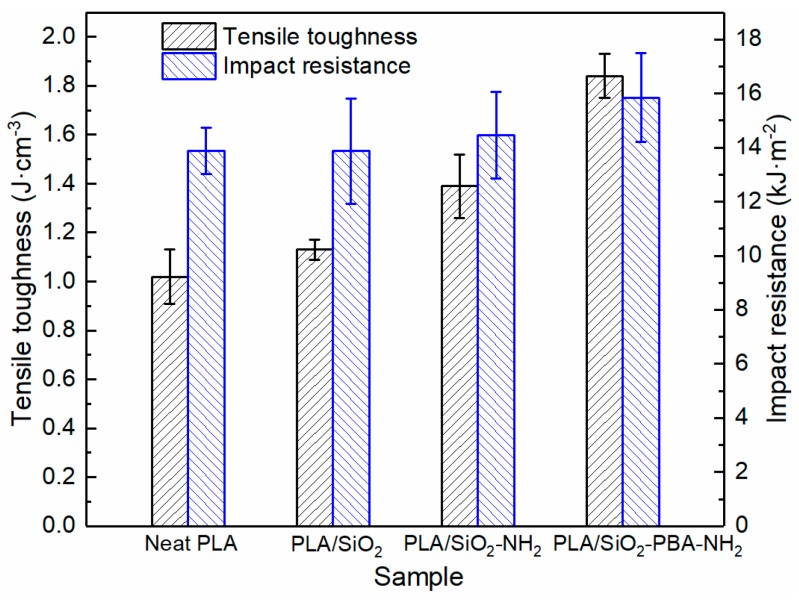
Tensile toughness and impact resistance of neat PLA and PLA nanocomposites.

Furthermore, the core–shell nanoparticle also exhibits a remarkable toughening effect on PLA, as shown in Figure 6. With the incorporation of SiO_2_-PBA-NH_2_ nanoparticles, the toughness of the PLA nanocomposite improves 80.4% in comparison with that of neat PLA. This may result from the good interfacial adhesion and the crack tip pinning effect. Moreover, from the Figure 6, the impact strength of the PLA/SiO_2_-PBA-NH_2_ nanocomposite is improved to 15.85 kJ·m^−2^ from 13.88 kJ·m^−2^, an increment of 14.2%, with only 1 wt. % loading of the core–shell nanofiller. The other rigid nanoparticles show nearly no enhancement of the impact resistance of PLA. This indicates that the insertion of a flexible PBA layer can absorb more impact energy.

Previous research has revealed that the elongation at break of PLA composites was enhanced at the expense of their strength and stiffness [25,26]. However, in our study, it is demonstrated that only a low loading of core–shell nanoparticles can achieve the balance of strengthening and toughening resulting from the synergistic effect between the rigid core and flexible shell.

### 4.3. Toughening Mechanisms of Nanoparticles

**Figure 7:**
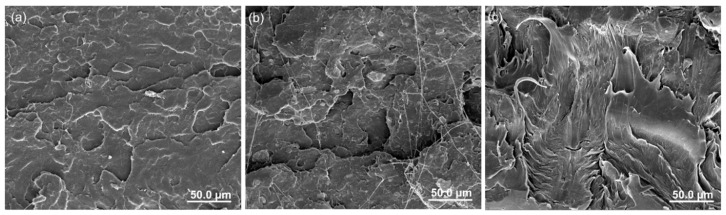
Scanning electron microscopy (SEM) fracture surface morphologies of PLA nanocomposites after tensile testing. (**a**) Neat PLA, (**b**) PLA/SiO_2_-NH_2_, (**c**) PLA/SiO_2_-PBA-NH_2_.

The fracture surface morphologies of PLA nanocomposite after tensile testing are displayed in Figure 7. The neat PLA exhibits a relatively smooth fracture surface in Figure 7a, in which some shallow cracks indicate brittle fracture behavior. As shown in Figure 7b, the fracture surface of the SiO_2_-NH_2_ nanoparticle-modified PLA composite is much rougher than that of neat PLA, and some distinct micro-cracks appeared, which indicates that the added rigid nanoparticle can induce the effect of crack pinning and deflecting. More notably, when incorporating the core–shell nanoparticle into the PLA matrix, obvious plastic deformation occurred in the fractured surface of nanocomposite, presenting a typical ductile fracture behavior, which dissipated more energy to improve the fracture toughness. This observation can be ascribed to the fact that the rubber shell of the core–shell nanoparticle initiates the shear yielding of the polymer.

**Figure 8:**
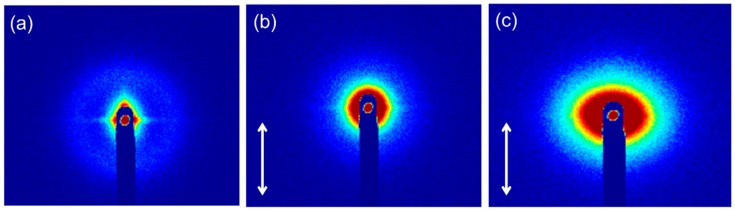
Two-dimensional small-angle X-ray scattering (SAXS) scattering patterns of (**a**) neat PLA before tensile testing, (**b**) PLA/SiO_2_ and (**c**) PLA/SiO_2_-PBA-NH_2_ after tensile testing (the double-headed arrows indicate the tensile direction).

SAXS is considered to be an effective technique for the local deformation and submicroscopic inhomogeneity characterization of samples. The scattering intensity increases at the equator (perpendicular to the tensile direction) due to the appearance of the cavitation. Figure 8 shows the 2D-SAXS scattering patterns of neat PLA before tensile testing and PLA nanocomposites reinforced by raw silica and core–shell nanoparticles after tensile tests. Primarily, the unformed sample of neat PLA exhibits low intensity (Figure 8a). After tensile testing, the deformed sample of PLA/SiO_2_ showed isotropic 2D scattering patterns and increased intensity (Figure 8b), which indicates the formation of micro-cracks, coinciding with the features displayed in SEM images. Notably, as seen in Figure 8c, the SAXS scattering pattern of PLA reinforced by core–shell nanoparticles after deformation presents an elliptical shape and a significant intensity increase in the equatorial direction. Accordingly, under tension, the cavitation appears in the flexible shell of core–shell nanoparticles [19], which promotes the initiation and growth of cavities around matrix. These cavities are oriented along the tensile direction and thus lead to an ellipsoidal scattering pattern in the equatorial direction, exhibited in the 2D SAXS image of deformed PLA/SiO_2_-PBA-NH_2_. Consequently, the growth of cavities releases the triaxial stress, which initiates the shear yielding of PLA and subsequently impedes the crack propagation [27], coinciding with the SEM images.

## 5. Numerical Analysis and Comparison with Experimental Results

### 5.1. Mechanical Behavior and Damage Process of PLA/SiO_2_ Composite

**Figure 9:**
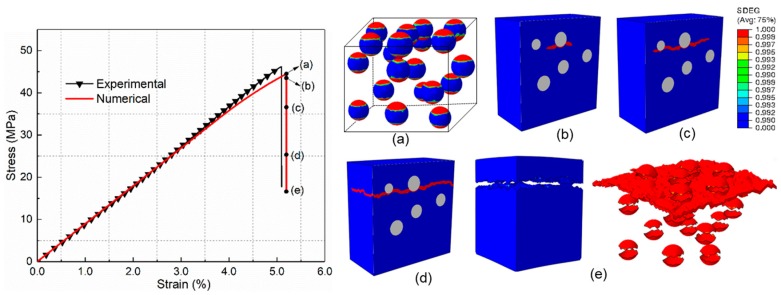
Progressive damage process of PLA/SiO_2_ under uniaxial tension for the multi-particle model. (**a**) Interfacial debonding (**b**) Matrix crack initiation (**c**) and (**d**) Matrix crack propagation (**e**) Debonding regions and matrix crack.

For comparison with the core–shell particle-reinforced composite, the mechanical behavior and damage process of composites incorporated by the raw silica are investigated first. Figure 9 shows the tensile stress–strain curves of raw silica-reinforced composite obtained by the experiment and numerical simulation. It can be seen that the numerical result is in good agreement with the experimental results. The damage initiation and evolution process of PLA/SiO_2_ under uniaxial tension are presented in Figure 9a–e (SDEG, scalar stiffness degradation variable). Figure 9a illustrates that the main damage mechanism at maximum stress is interfacial debonding. Each particle debonds from the surrounding matrix. After the interface debonding, the maximum microscopic hydrostatic stress concentrates mainly at the tip of the debonding zone, which induces matrix cracking (Figure 9b). Then, the matrix cracking propagates to the nearby debonding particles and extends in the matrix, as presented in Figure 9c,d. Finally, as can be seen from Figure 9e, the interfacial debonding at different locations is linked by the matrix cracks throughout the whole RVE, which causes the ultimate failure of the composite.

**Figure 10:**
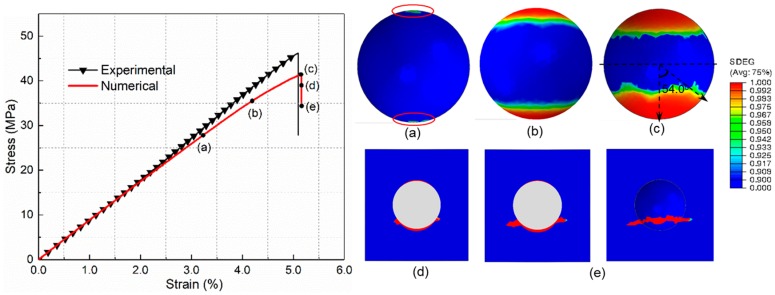
Progressive damage process of PLA/SiO_2_ under uniaxial tension for the single-particle model. (**a**) Interfacial debonding initiation. (**b**,**c**) Debonding propagation. (**d**) Matrix crack initiation. (**e**) Matrix crack propagation.

To illustrate the local mechanical behavior and progressive failure process more clearly, the single-particle micromechanics model is presented. It can be found from Figure 10 that the softening behavior of the stress–strain curve induced by the interfacial debonding is more distinct in the single-particle model. Hence, the ultimate tensile strength obtained from the single-particle model is slightly lower than that from the multi-particle model, which is accordance with Llorca and Segurado’s results [20]. As shown in Figure 10a, the interfacial debonding initiates at the poles of the particle in the direction of loading at a strain of 3.2%. With the strain increasing, the debonding damage propagates progressively from two poles to the equator (Figure 10b). Subsequently, the rate of propagation decreases until the debonding damage process ceases, which is attributed to the formed compressive regions in the plane perpendicular to the loading direction [28]. As exhibited in Figure 10c, the ultimate debonding angle measured from the pole to the front of the debonded regions is about 54°, which is in agreement with the conclusion reported by Cho et al. [15]. As the tensile deformation increases (Figure 10d), microcracks of the matrix are initiated at the overlapping regions of the interfacial debonding tip and compressive region, which is also illustrated by Tsui et al. [29]. After the initiation, in Figure 10e, the matrix cracks extend around the debonding region perpendicular to the tensile direction.

**Figure 11:**
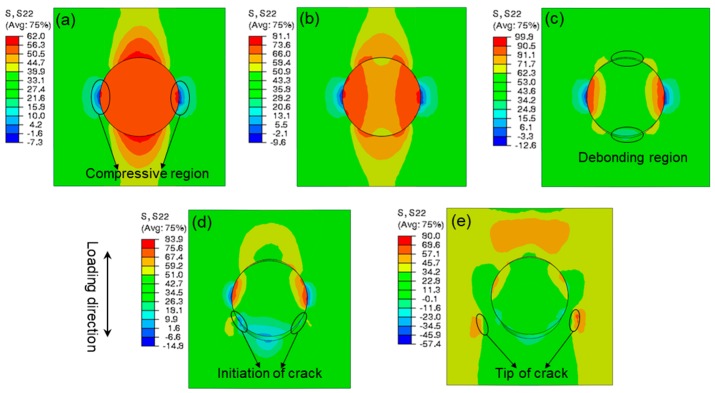
Distribution of normal stress along the loading axis of PLA/SiO_2_ at the stage of (**a**) the initiation of interfacial debonding, (**b**) propagation of debonding (**c**), cessation of debonding, (**d**) initiation of matrix cracking (**e**), propagation of matrix cracking.

Figure 11 presents the stress distribution of the single-particle model in the tensile direction. It can be seen from Figure 11a that, under tensile loading, the rigid silica with a high modulus has a primary load-bearing capacity. The maximal tensile stress of the material is concentrated in the polar zones along the loading axis. As the tensile stress in the interface increases to the interfacial strength, the interfacial debonding initiates. Meanwhile, the compressive regions appear in the equator perpendicular to the loading direction. In addition to stretching, the interfacial debonding proceeds from the polar zone to the equator (Figure 11b). As the interfacial debonding ceases, resulting from the impediment of compressive stress, the load-bearing capacity of the rigid particle begins to decline, which results in the tensile stress of the whole model reaching its maximum, and cavities appear at the polar zones (Figure 11c). Subsequently, as the cavities extend to the equator, the maximal microscopic tensile stress in the matrix concentrates at the tips of cavities. When this tip proceeds to the compressive stress region, matrix cracking is initiated, as presented in Figure 11d. Eventually, owing to the prevention of front compressive stress, the matrix crack propagates along the direction perpendicular to the tensile axis (Figure 11e), and the stress in the tip of crack stays at maximum.

### 5.2. Mechanical Behavior and Damage Process of PLA/SiO_2_-PBA-NH_2_ Composite

**Figure 12:**
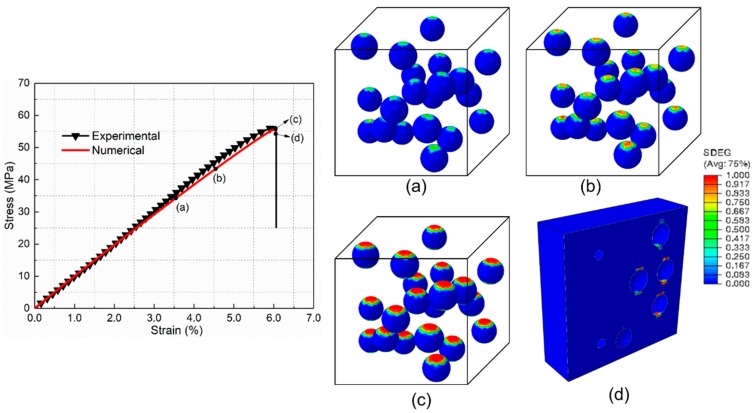
Progressive damage process of PLA/SiO_2_-PBA-NH_2_ under uniaxial tension for the multi-particle model. (**a**,**b**) Initiation and propagation of rubber shell cavitation. (**c**) Cavitation propagation cease. (**d**) Plastic yielding of matrix at poles.

**Figure 13:**
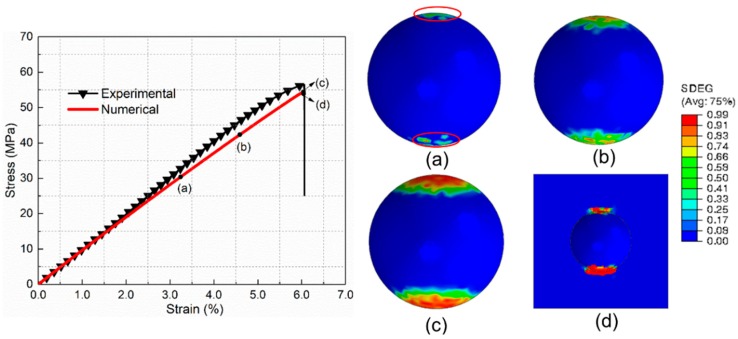
Progressive damage process of PLA/SiO_2_-PBA-NH_2_ under uniaxial tension for the single-particle model. (**a**,**b**) Initiation and propagation of rubber shell cavitation. (**c**) Cavitation propagation cease. (**d**) Plastic yielding of matrix at poles.

The progressive damage process of the core–shell nanoparticle (SiO_2_-PBA-NH_2_)-reinforced nanocomposite presented in the multi-particle model (Figure 12) and single-particle model (Figure 13) are similar; thus, we illustrate the damage process from the single-particle model shown in Figure 13. Under uniaxial tension, the cavitation of the polymer shell is initiated at the two poles first (Figure 13a). Unlike the interfacial debonding of the rigid particle-reinforced composite (Figure 12b), the damage factor of the cohesive element in the core–shell nanofiller-reinforced composite increases slowly, which is attributed to the deformation of the flexible shell. Afterwards, the cavitation of the flexible shell extends progressively to the equator (Figure 13b). As the deformation increases, the propagation rate of cavitation decreases until the cavitation ceases, which is due to the formation of compressive regions in the polymer shell with a high Poisson’s ratio. Subsequently, the formed cavitation damage further develops (Figure 13c), which releases the stress triaxiality acting in the matrix and thereby promotes the shear yielding of the matrix. Consequently, as presented in Figure 13d, the deformation damage of the matrix appears at two poles linked to the nanoparticles.

**Figure 14:**
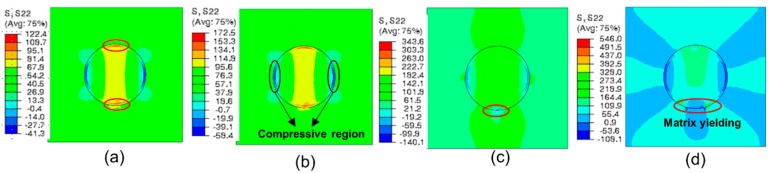
The stress distribution of PLA/SiO_2_-PBA-NH_2_ under uniaxial tension: (**a**) initiation and (**b**) propagation of polymer shell cavitation; (**c**) initiation and (**d**) propagation of matrix yielding.

Figure 14 presents the stress distribution of PLA/SiO_2_-PBA-NH_2_ under tensile loading, which shows a significant difference with that of PLA/SiO_2_. The tensile stress is concentrated in two poles along the tensile direction, which induces the cavitation of the soft polymer shell in the core–shell nanoparticle; meanwhile, the compressive regions appear in the equator perpendicular to the tensile direction (Figure 14a). Subsequently, the cavitation further propagates, which releases the stress triaxiality around the matrix and thus initiates the shear yielding of the matrix, as shown in Figure 14c. At present, the stress previously concentrated in the nanofiller expands to the matrix. As the matrix yielding propagates, the area of stress extension gradually increases (Figure 14d); meanwhile, the specimen unloads.

## 6. Conclusions

A core–shell nanoparticle consisting of a rigid silica core and flexible polymer shell has been synthesized in this study to enhance the strength and toughness of PLA. Both experimental and numerical investigations were performed to determine the effect of core–shell nanoparticles on the mechanical behavior and damage process of PLA. The main conclusions are summarized as follows.

(1) The experimental results showed that the incorporation of core–shell nanofiller is an excellent strategy to simultaneously improve the strength and toughness of biodegradable poly (lactic acid), as the tensile, flexural properties and impact resistance of PLA were improved; in particular, the tensile toughness and elongation at break of PLA were enhanced. Furthermore, the proposed micromechanical model can be used to illustrate the experimental behaviors of particle-reinforced composites.
(2) From the experimental observation combined with the numerical analysis, it was shown that for the rigid particle reinforced composite, the interfacial debonding and subsequent matrix cracking predominantly occur during the tension. Notably, as the flexible polymer layer was covered on the rigid particle, the failure process of the composite transforms to the cavitation of the polymer shell and induces matrix shear yielding, which plays a vital role in the toughening of the polymer.

## Figures and Tables

**Table 1:** The mechanical properties of Poly (lactic acid) (PLA) and PLA nanocomposites.

Sample	Tensile Properties				Flexural Properties		Impact Resistance
	Tensile Strength (MPa)	Young’s Modulus (GPa)	Elongation at Break (%)	Toughness (J·cm^−3^)	Flexural Strength (MPa)	Flexural Modulus (GPa)	(kJ·m^−2^)
Neat PLA	43.1 ± 1.4	0.94 ± 0.06	4.64 ± 0.39	1.02 ± 0.11	63.3 ± 4.3	2.42 ± 0.25	13.88 ± 0.86
PLA/SiO_2_	44.9 ± 1.1	0.87 ± 0.03	5.04 ± 0.09	1.13 ± 0.04	71.2 ± 3.2	2.28 ± 0.22	13.87 ± 1.95
PLA/SiO_2_-NH_2_	50.7 ± 2.3	0.91 ± 0.02	5.46 ± 0.25	1.39 ± 0.13	75.0 ± 4.4	2.41 ± 0.01	14.46 ± 1.60
PLA/SiO_2_-PBA-NH_2_	59.0 ± 2.2	1.02 ± 0.02	6.01 ± 0.11	1.84 ± 0.09	102.4 ± 7.1	3.24 ± 0.17	15.85 ± 1.65

## Supplementary Materials

The following are available online at https://www.mdpi.com/1996-1944/12/16/2510/s1, Figure S1: FTIR spectra of SiO_2_ and SiO_2_-PBA-NH_2_ nanoparticle, Figure S2: 1HNMR spectra of core-shell nanoparticle Figure S3: (a) Mechanical and damage behavior of matrix (b) Traction–separation law of cohesive element.

## References

[1] Kakroodi A.R., Kazemi Y., Ding W., Ameli A., Park C.B. (2015). Poly (lactic acid)-based in situ microfibrillar composites with enhanced crystallization kinetics, mechanical properties, rheological behavior, and foaming ability. Biomacromolecules.

[2] Nofar M., Park C.B. (2014). Poly (lactic acid) foaming. Prog. Polym. Sci..

[3] Gross R.A., Kalra B. (2002). Biodegradable polymers for the environment. Science.

[4] Nampoothiri K.M., Nair N.R., John R.P. (2010). An overview of the recent developments in polylactide (PLA) research. Bioresour. Technol..

[5] Joziasse C.A., Veenstra H., Grijpma D.W., Pennings A.J. (1996). On the chain stiffness of poly (lactide) s. Macromol. Chem. Phys..

[6] Zhang C., Wang W., Huang Y., Pan Y., Jiang L., Dan Y., Peng Z. (2013). Thermal, mechanical and rheological properties of polylactide toughened by expoxidized natural rubber. Mater. Des..

[7] Bitinis N., Verdejo R., Cassagnau P., Lopez-Manchado M.A. (2011). Structure and properties of polylactide/natural rubber blends. Mater. Chem. Phys..

[8] Jiang L., Zhang J., Wolcott M.P. (2007). Comparison of polylactide/nano-sized calcium carbonate and polylactide/montmorillonite composites: Reinforcing effects and toughening mechanisms. Polymer.

[9] Petchwattana N., Covavisaruch S., Petthai S. (2014). Influence of talc particle size and content on crystallization behavior, mechanical properties and morphology of poly (lactic acid). Polym. Bull..

[10] Fukushima K., Tabuani D., Arena M., Gennari M., Camino G. (2013). Effect of clay type and loading on thermal, mechanical properties and biodegradation of poly (lactic acid) nanocomposites. React. Funct. Polym..

[11] Thitsartarn W., Fan X., Sun Y., Yeo J.C.C., Yuan D., He C. (2015). Simultaneous enhancement of strength and toughness of epoxy using POSS-Rubber core–shell nanoparticles. Compos. Sci. Technol..

[12] Liu S., Fan X., He C. (2016). Improving the fracture toughness of epoxy with nanosilica-rubber core-shell nanoparticles. Compos. Sci. Technol..

[13] Williams J.J., Segurado J., LLorca J., Chawla N. (2012). Three dimensional (3D) microstructure-based modeling of interfacial decohesion in particle reinforced metal matrix composites. Mater. Sci. Eng. A.

[14] Meng Q., Wang Z. (2015). Prediction of interfacial strength and failure mechanisms in particle-reinforced metal-matrix composites based on a micromechanical model. Eng. Fract. Mech..

[15] Cho J., Joshi M.S., Sun C.T. (2006). Effect of inclusion size on mechanical properties of polymeric composites with micro and nano particles. Compos. Sci. Technol..

[16] Tsui C.P., Tang C.Y., Fan J.P., Xie X.L. (2004). Prediction for initiation of debonding damage and tensile stress–strain relation of glass-bead-filled modified polyphenylene oxide. Int. J. Mech. Sci..

[17] Li S., McCarthy S. (1999). Influence of crystallinity and stereochemistry on the enzymatic degradation of poly (lactide) s. Macromolecules.

[18] Goudarzi T., Spring D.W., Paulino G.H., Lopez-Pamies O. (2015). Filled elastomers: A theory of filler reinforcement based on hydrodynamic and interphasial effects. J. Mech. Phys. Solids.

[19] Ke Z., Shi D., Yin J., Li R.K., Mai Y.W. (2008). Facile method of preparing supertough polyamide 6 with low rubber content. Macromolecules.

[20] LLorca J., Segurado J. (2004). Three-dimensional multiparticle cell simulations of deformation and damage in sphere-reinforced composites. Mater. Sci. Eng. A.

[21] Nofar M., Tabatabaei A., Park C.B. (2013). Effects of nano-/micro-sized additives on the crystallization behaviors of PLA and PLA/CO_2_ mixtures. Polymer.

[22] Domun N., Hadavinia H., Zhang T., Sainsbury T., Liaghat G.H., Vahid S. (2015). Improving the fracture toughness and the strength of epoxy using nanomaterials–a review of the current status. Nanoscale.

[23] Yang H., Li F., Shan C., Han D., Zhang Q., Niu L., Ivaska A. (2009). Covalent functionalization of chemically converted graphene sheets via silane and its reinforcement. J. Mater. Chem..

[24] Li X., Lei B., Lin Z., Huang L., Tan S., Cai X. (2014). The utilization of bamboo charcoal enhances wood plastic composites with excellent mechanical and thermal properties. Mater. Des..

[25] Herrera N., Mathew A.P., Oksman K. (2015). Plasticized polylactic acid/cellulose nanocomposites prepared using melt-extrusion and liquid feeding: Mechanical, thermal and optical properties. Compos. Sci. Technol..

[26] Choi K.M., Choi M.C., Han D.H., Park T.S., Ha C.S. (2013). Plasticization of poly (lactic acid)(PLA) through chemical grafting of poly (ethylene glycol)(PEG) via in situ reactive blending. Eur. Polym. J..

[27] Dompas D., Groeninckx G. (1994). Toughening behaviour of rubber-modified thermoplastic polymers involving very small rubber particles: 1. A criterion for internal rubber cavitation. Polymer.

[28] Matouš K., Geubelle P.H. (2006). Multiscale modelling of particle debonding in reinforced elastomers subjected to finite deformations. Int. J. Numer. Methods Eng..

[29] Tsui C.P., Chen D.Z., Tang C.Y., Uskokovic P.S., Fan J.P., Xie X.L. (2006). Prediction for debonding damage process and effective elastic properties of glass-bead-filled modified polyphenylene oxide. Compos. Sci. Technol..

