# Supplementary material for: The Strengthening and Toughening of Biodegradable Poly (Lactic Acid) Using the SiO2-PBA Core–Shell Nanoparticle
Source: Materials (Basel). 2019 Aug 7;12(16):2510. doi: 10.3390/ma12162510 (PMC6720591; doi:10.3390/ma12162510)
Supplement: Supplementary file 1 [file materials-12-02510-s001.pdf]

materials

Supplementary Information

The Strengthening and Toughening of Biodegradable Poly (Lactic Acid) Using the SiO_2-PBA Core–Shell Nanoparticle

Hailing He [1], Yuezhao Pang [1], Zhiwei Duan [2], Na Luo[1] and Zhenqing Wang [1,*]

[1] College of Aerospace and Civil Engineering, Harbin Engineering University, Harbin 150001, China
[2] Institute of Fluid Physics, China Academy of Engineering Physics, Mianyang, Sichuan 621900, China
* Correspondence: wangzhenqing@hrbeu.edu.cn

Received: 10 July 2019; Accepted: 05 August 2019; Published: date

1. The Characterization of Core-Shell Nanoparticle

We characterized the graft polymerization of flexible polymer shell using the Fourier Transform Infrared Spectroscopy (FTIR), the result is presented in Figure S1. It can be seen that comparing with the FTIR spectrum of raw SiO_2, the spectrum of core-shell particle (SiO_2-PBA-NH_2) presented the new characteristic peaks at 1396 cm^{-1} from the C-N stretching vibration and at 1650 cm^{-1} from the C=O of acylamide, which demonstrates the 2-bromoisobutyryl bromide being initiated to the silica [1]. The adsorption peak at 1732 cm^{-1} was the characteristic of C=O group from the PBA. Meanwhile, the peaks at 2875 cm^{-1}, 1382 cm^{-1} and 1433 cm^{-1} from the C-H vibration indicated the presence of methyl group. The peak at 2934 cm^{-1} and 1469 cm^{-1} was attributed to the C-H stretching and bending vibration of methylene. All of these indicated that the PBA was grafted on the silica surface successfully [2]. The stretching vibration of C-S at 694 $cm^{-1}$and the bending vibration of NH_2 at 1533 cm^{-1} revealed that the amine groups were bonded to the terminal of polymer shell.

Further to confirm this characteristic, we analyzed the ^{1}H nuclear magnetic resonance (^{1}HNMR) spectroscopy of core-shell nanoparticle using $CDCl_3$ as the solvent, as shown in Figure S2. It is found that the chemical shifts at 1.8 ppm (a), 4.1 ppm (c), 1.6 ppm (d) and 1.4 ppm (e) are different methylene protons of PBA units. And the signal at δ~ 0.9 ppm (f) and δ~ 2.3 ppm (b) was assigned to the methyl and methenyl protons of PBA, respectively. These chemical shifts also indicated that the successful grafting of the PBA polymer onto silica nanoparticle.

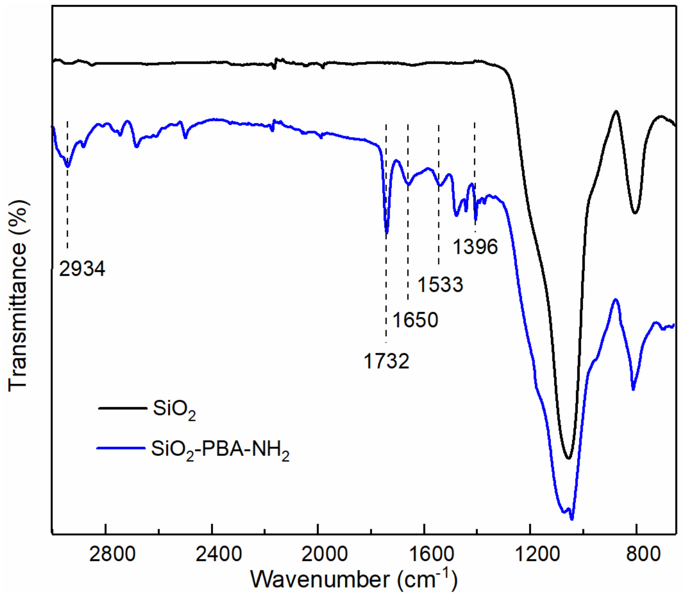

Figure S1. FTIR spectra of SiO_2 and SiO_2-PBA-NH_2 nanoparticle.

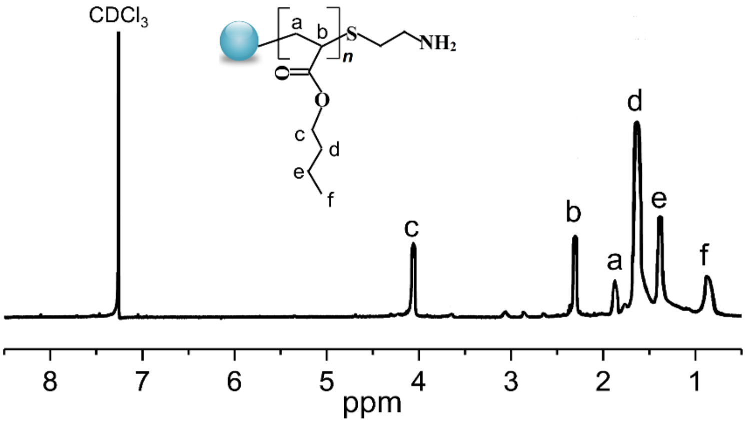

Figure S2. ^{1}HNMR spectra of core-shell nanoparticle. 1.8 ppm (**a**), 2.3 ppm (**b**), 4.1 ppm (**c**), 1.6 ppm (**d**) and 1.4 ppm (**e**), 0.9 ppm (**f**).

2. Material models

2.1. Material Properties of Inclusion

The spherical silica particle is considered as elastic and isotropic solids characterized by E_p=70 GPa, υ_p=0.17 [3], and the failure of silica is not accounted in this paper.

In core-shell particle reinforced PLA model, the poly (n-butyl acrylate) shell is set as an isotropic hyperelastic material, which exhibits very large strain and a strong non-linear stress-strain behavior. Hyperelastic material model is derived from a strain-energy density function $W(\lambda_1, \lambda_2, \lambda_3)$, where λ_1, λ_2 and λ_3 are three principal stretches. In this study, the first order Mooney–Rivlin model is implemented to describe the stress-strain response of rubbery shell, which can be expressed as [4]:

$$W = C_{10}(I_1 - 3) + C_{01}(I_2 - 3) \quad (1)$$

where W is the strain energy density, C_{10} and C_{01} are the temperature-dependent material parameters, I_1 and I_2 are first and second deviatoric strain invariants defined as:

$$\begin{cases} I_1 = \lambda_1^2 + \lambda_2^2 + \lambda_3^2 \\ I_2 = (\lambda_1\lambda_2)^2 + (\lambda_2\lambda_3)^2 + (\lambda_1\lambda_3)^2 \end{cases} \quad (2)$$

In uniaxial tension, the stress-strain laws of Mooney–Rivlin model can be obtained from its strain-energy function W:

$$\sigma = 2(\lambda - \lambda^{-2})(C_{10}\lambda + C_{01}) \quad (3)$$

where σ is the applied stress, λ is the extension ratio. According to the uniaxial tensile tests of soft poly (n-butyl acrylate) networks implemented by Cui et al. [5], the model coefficients of C_{10} and C_{01} are given as 0.1 and 0.03 MPa, respectively.

2.2. Matrix Material and Damage

The polymer matrix shows a rather brittle fracture behavior in uniaxial tension, but it shows considerable plastic deformation in compression and pure shear [6–8]. Thus, the matrix is assumed to behave as an elastic-plastic solid, moreover, it is found that the behavior of polymers is sensitive to the hydrostatic pressure [9]. These characteristics can be captured via Mohr-Coulomb or Drucker–Prager yield criterion [10]. However, the Drucker–Prager criterion with circular cone yield surface is more attractive in the numerical implementation than the Mohr–Coulomb criterion with hexagonal cone yield surface, since it has a continuously varying normal [11]. Furthermore, the parameters of Drucker–Prager model can be obtained conveniently via the conversion of the Mohr–Coulomb

model. So, the extended linear Drucker–Prager model is employed to the matrix in this study, which can be expressed as:

$$F = t - p\tan\beta - d = 0, t = \frac{1}{2}q\left[1 + \frac{1}{k} - \left(1 - \frac{1}{k}\right)\left(\frac{r}{q}\right)^3\right] \quad (4)$$

where p is the hydrostatic stress, q is the Mises equivalent stress, r is the third invariant of deviatoric stress, β is the slope of the linear yield surface in the $p-t$ stress plane, d is the cohesion of the material, and k is the ratio of the yield stress in triaxial tension to the yield stress in triaxial compression.

The slope of β and yield stress ration of k can be determined by the following equations:

$$\tan\beta = \frac{6\sin\phi}{3-\sin\phi}, \; k = \frac{3-\sin\phi}{3+\sin\phi} \quad (5)$$

where ϕ is the internal friction angle of material, which is generally set as 15° to represent the polymer matrix [12], so it can obtain β=29.5°, k=0.84.

The matrix cohesion of d can be defined by the uniaxial tension strength σ_t:

$$d = \left(\frac{1}{k} + \frac{1}{3}\tan\beta\right)\sigma_t \quad (6)$$

From the Equation (6), it gives d =59.4 MPa. Besides, the elastic properties of matrix are determined experimentally, E_m= 934 MPa and $\upsilon_m = 0.36$.

The localized inelastic deformation of matrix can be induced by the debonding of rigid particle and the cavitation of flexible shell [13,14]. The micro-voids and micro-cracks caused by this can lead to the degradation of materials. To capture the process of the damage, the ductile criterion is used to predict the damage initiation of matrix materials. The model assumes that the equivalent plastic strain at the onset of damage, $\bar{\varepsilon}_D^{pl}$, is a function of stress triaxiality η, where $\eta = -p/q$. The stress triaxiality is a measurement of the triaxial stress state, η takes the value of 1/3 under uniaxial tension. The criterion for damage initiation is met when the following condition is satisfied:

$$\omega_D = \int \frac{d\bar{\varepsilon}^{pl}}{\bar{\varepsilon}_D^{pl}} = 1 \quad (7)$$

where ω_D is a state variable that increases monotonically with the plastic deformation.

After the onset of damage, the damage evolution is performed by a progressive failure procedure, the stress-strain behavior of which is illustrated in Figure S3a. The solid curve in the figure represents the damaged stress-strain response, while the dashed curve is the response in the absence of the damage. The damage manifests itself in two forms: softening of yield stress and degradation of elasticity, which can be characterized by the damage variable, D, which increases with the evolution of damage according to:

$$D = \frac{L\dot{\bar{\varepsilon}}_f^{pl}}{\bar{u}_f^{pl}} = \frac{\dot{\bar{u}}_f^{pl}}{\bar{u}_f^{pl}} \quad (8)$$

where L is the characteristic length of the element, $\bar{\varepsilon}^{pl}$, $\bar{u}^{pl}$ are the equivalent plastic strain and displacement, respectively. Before damage initiation, $\dot{\bar{u}}^{pl} = 0$, after damage initiation, $\dot{\bar{u}}^{pl} = L\dot{\bar{\varepsilon}}^{pl}$. $\bar{u}_f^{pl}$ is the equivalent plastic displacement at failure, which can be calculated as $\bar{u}_f^{pl} = 2G_f/\sigma_{y0}$. σ_{y0} is the yield stress at the time of reaching the failure criterion. G_f is the fracture energy per unit area, which is given as:

$$G_f = \int_{\bar{\varepsilon}_0^{pl}}^{\bar{\varepsilon}_f^{pl}} L\sigma_y \, d\bar{\varepsilon}^{pl} = \int_0^{\bar{u}_f^{pl}} \sigma_y \, d\bar{u}^{pl} \quad (9)$$

where $\bar{\varepsilon}_0^{pl}$ and $\bar{\varepsilon}_f^{pl}$ are the equivalent plastic strains at the onset of damage (D = 0) and at final failure (D = 1), respectively.

The tensile strength of neat poly (lactic acid) is 43 MPa measured by our experiment. The experiment show that the final failure is brittle under uniaxial tension, thus the equivalent plastic displacement at failure is given as a very small value of 5 ×10^{-5} μm.

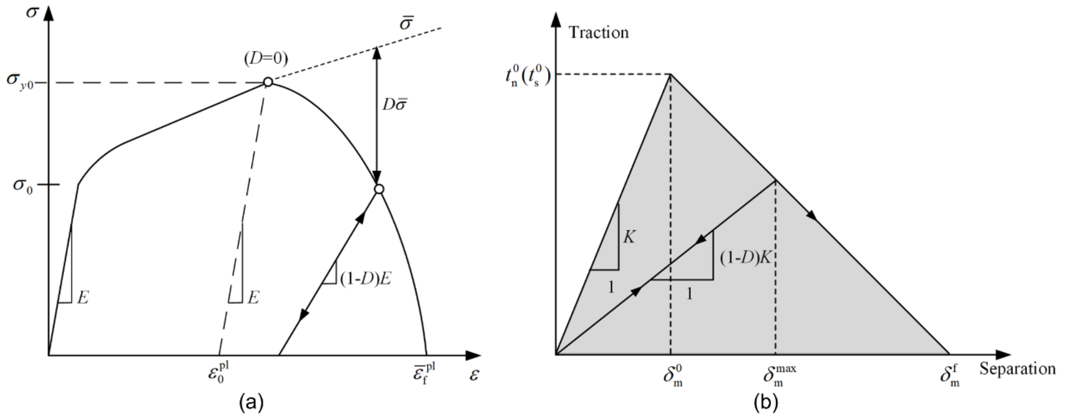

Figure S3. (**a**) Mechanical and damage behavior of matrix (**b**) Traction–separation law of cohesive element.

2.3. Cohesive zone model

The progressive debonding of raw silica and cavitation of flexible polymer shell between inclusion and matrix are simulated by cohesive zone model. The mechanical behavior of interface can be expressed via a traction-separation law which relates the separation displacement across the cohesive elements with the traction vector acting on it. The constitutive response of cohesive element applied in this study is defined in terms of a bi-liner traction-separation law, as presented in Figure S3b. The initial linear response in absence of damage can be written as:

$$t_n = K\delta_n, t_s = K\delta_s, t_t = K\delta_t \qquad (10)$$

where the traction stress vector, ***t***, consists of three components, t_n, t_s, t_t, which represent the normal and two shear traction, respectively. The corresponding separations displacement are denoted by δ_n, δ_s, δ_t. The elastic stiffness of the interface element, *K*, is set as 10^8 GPa/mm in this paper to ensure the displacement continuity around the particle at interface in absence of damage [15].

The degradation process of interface element will be initiated when its stresses and/or strains satisfy certain damage initiation criteria. Here, the quadratic stress failure criterion is considered for the damage onset of cohesive element, which can be represented as:

$$\left\{\frac{\langle t_n \rangle}{t_n^0}\right\}^2 + \left\{\frac{t_s}{t_s^0}\right\}^2 + \left\{\frac{t_t}{t_t^0}\right\}^2 = 1 \qquad (11)$$

where $\langle\ \rangle$ is the Macaulay brackets, which return the argument if positive and zero otherwise, to impede the development of damage when the interface is under compression. t_n^0, t_s^0 and t_t^0 represent the peak values of the nominal stress when the deformation is either purely normal to the interface or purely in the first or the second shear direction, respectively. The strength of interface is assumed to be equal to the cohesion of matrix [10], $t_n^0 = t_s^0 = t_t^0 = d$=59.4 MPa.

After the initiation of damage, the scalar damage variable, *D*, monotonically evolves from 0 to 1 upon further loading. The stress components of the traction-separation model are reduced by the damage according to:

$$t_n = \begin{cases} (1 - D)K\delta_n & \text{if } \delta_n > 0, \\ K\delta_n & \text{otherwise} \end{cases} \tag{12}$$

$$t_s = (1 - D)K\delta_s \tag{13}$$

$$t_t = (1 - D)K\delta_t \tag{14}$$

To describe the evolution of damage under a combination of normal and shear deformation across the interface, an effective displacement, δ_{m} is introduced:

$$\delta_m = \sqrt{\delta_n^2 + \delta_s^2 + \delta_t^2} \tag{15}$$

For computational convenience, the linear form of damage evolution law is based on effective displacement [16]. The evolution of the damage variable D can be expressed as:

$$D = \frac{\delta_m^f(\delta_m^{\max} - \delta_m^0)}{\delta_m^{\max}(\delta_m^f - \delta_m^0)} \tag{16}$$

where δ_{m}^0 *and* $\delta_{\mathrm{m}}^{\mathrm{f}}$ are the effective displacement at onset of damage (D = 0) and complete failure (D = 1), respectively. $\delta_{\mathrm{m}}^{\max}$ is related to the maximum value of the effective displacement attained during the loading history (as shown in Figure S3b). In this study, the damage evolution is defined based on the displacement at failure, which is set as 5 ×10^{-4} μm.

References

1. Zhan, X.; Yan, Y.; Zhang, Q.; Chen, F. A novel superhydrophobic hybrid nanocomposite material prepared by surface-initiated AGET ATRP and its anti-icing properties. Journal of Materials Chemistry A. **2014**, 2, 9390-9399.
2. Ren, Y., Zhou, G., Cao, P. Preparations and properties of a tunable void with shell thickness SiO2@ SiO2 core–shell structures via activators generated by electron transfer for atom transfer radical polymerization. Solid State Sciences. **2016**, 52, 154-162.
3. Dittanet, P.; & Pearson, R. A. Effect of bimodal particle size distributions on the toughening mechanisms in silica nanoparticle filled epoxy resin. Polymer. **2013**, 54, 1832-1845.
4. Sasso, M.; Palmieri, G.; Chiappini, G.; & Amodio, D. Characterization of hyperelastic rubber-like materials by biaxial and uniaxial stretching tests based on optical methods. Polymer Testing. **2008**, 27, 995-1004.
5. Cui, J.; Kratz, K.; Hiebl, B.; Jung, F.; Lendlein, A. Soft poly (n-butyl acrylate) networks with tailored mechanical properties designed as substrates for in vitro models. Polymers for Advanced Technologies. **2011**, 22, 126-132.
6. Robles, E.; Urruzola, I.; Labidi, J.; & Serrano, L. Surface-modified nano-cellulose as reinforcement in poly (lactic acid) to conform new composites. Industrial Crops and Products. **2015**, 71, 44-53.
7. Smit, T. H.; Engels, T. A.; Söntjens, S. H.; & Govaert, L. E. Time-dependent failure in load-bearing polymers: a potential hazard in structural applications of polylactides. Journal of Materials Science: Materials in Medicine. **2010**, 21, 871-878.
8. Fiedler, B.; Hojo, M.; Ochiai, S.; Schulte, K.; & Ando, M. Failure behavior of an epoxy matrix under different kinds of static loading. Composites Science and Technology. **2001**, 61, 1615-1624.
9. Asp, L. E.; Berglund, L. A.; & Gudmundson, P. Effects of a composite-like stress state on the fracture of epoxies. Composites science and technology. **1995**, 53, 27-37.
10. Yang, L.; Yan, Y.; Liu, Y.; & Ran, Z. Microscopic failure mechanisms of fiber-reinforced polymer composites under transverse tension and compression. Composites Science and Technology. **2012**, 72, 1818-1825.
11. Romanowicz, M. A numerical approach for predicting the failure locus of fiber reinforced composites under combined transverse compression and axial tension. Computational Materials Science. **2012**, 51, 7-12.
12. González, C.; & LLorca, J. Mechanical behavior of unidirectional fiber-reinforced polymers under transverse compression: microscopic mechanisms and modeling. Composites Science and Technology. **2007**, 67, 2795-2806.
13. Gojny, F. H.; Wichmann, M. H.; Fiedler, B.; & Schulte, K. Influence of different carbon nanotubes on the mechanical properties of epoxy matrix composites–a comparative study. Composites Science and Technology. **2005**, 65, 2300-2313.

14. Guild, F. J.; & Kinloch, A. J. Modelling the properties of rubber-modified epoxy polymers. Journal of materials science. **1995**, 30, 1689-1697.
15. Canal, L. P.; Segurado, J.; & LLorca, J. Failure surface of epoxy-modified fiber-reinforced composites under transverse tension and out-of-plane shear. International journal of solids and structures. **2009**, 46, 2265-2274.
16. Camanho, P. P.; Davila, C. G.; & De Moura, M. F. Numerical simulation of mixed-mode progressive delamination in composite materials. Journal of composite materials. **2003**, 37, 1415-1438.

© 2019 by the authors. Submitted for possible open access publication under the terms and conditions of the Creative Commons Attribution (CC BY) license (http://creativecommons.org/licenses/by/4.0/).
